# How symptoms of simple acute infections affect the SSS-8 and SSD-12 as screening instruments for somatic symptom disorder in the primary care setting

**DOI:** 10.3389/fpsyt.2023.1114782

**Published:** 2023-04-17

**Authors:** Ying Zhang, David Baumeister, Mona Spanidis, Felicitas Engel, Sabrina Berens, Annika Gauss, Wolfgang Eich, Jonas Tesarz

**Affiliations:** ^1^Department of General Internal Medicine and Psychosomatics, University Hospital Heidelberg, Heidelberg University, Heidelberg, Germany; ^2^Department of Gastroenterology and Hepatology, University Hospital Heidelberg, Heidelberg, Germany

**Keywords:** simple acute infections, somatic symptom disorder, primary care, screening instruments, SSS-8, SSD-12

## Abstract

**Objective:**

Somatic symptom disorder (SSD) is one of the most common reasons for consultations in primary care, in addition to simple acute infections. Questionnaire-based screening instruments to identify patients at high risk of SSD are thus of great clinical relevance. Although screening instruments are frequently used, it is currently unclear to what extent they are influenced by the concurrent presence of simple acute infections. Therefore, this study aimed to investigate how symptoms of simple acute infections affect the two established questionnaires as screening instruments for somatic symptom disorder in the primary care setting.

**Methods:**

In our cross-sectional, multicenter design, a total of 1,000 patients in primary care practices were screened using the two most established SSD screening questionnaires, the 8-item Somatic Symptom Scale (SSS-8) and the 12-item Somatic Symptom Disorder—B Criteria Scale (SSD-12), followed by clinical assessment by the primary care physician.

**Results:**

A total of 140 patients with a simple acute infection (acute infection group, AIG) and 219 patients with chronic somatic symptoms (somatic symptom group, SSG) were included. The patients in the SSG showed higher total SSS-8 and SSD-12 scores than the patients in the AIG; however, the SSS-8 was more susceptible to changes triggered by symptoms of a simple acute infection than the SSD-12.

**Conclusion:**

These results suggest that the SSD-12 is less susceptible to symptoms of a simple acute infection. Its total score and corresponding cutoff value provide a more specific and thus less susceptible screening tool for identifying SSD in primary care.

## 1. Introduction

Somatic symptoms are the most common reason for patients to seek medical care. In many cases, somatic symptoms are related to somatic diseases and/or mental disorders. For example, simple acute infection is one of the most frequently encountered conditions in primary medical care (1, 2). According to Grobe, Steinmann and Szecseny (3), ~30% of German citizens in primary care were diagnosed with acute respiratory system infection in 2017. The somatic symptoms of simple acute infections vary widely. In a prospective online cohort study of the general population in England (4), 873 participants with infections reported a total of more than 40,590 symptoms, with 79% of participants reporting at least one symptom. These symptoms mainly related to the respiratory tract, the gastrointestinal system and the urinary tract (4). This variety of common symptoms suggests that patients with a simple acute infection could have a high symptom overlap with non-specific, functional, and somatoform (NFS) bodily complaint, which includes pain in various locations, impaired different organ functions (gastrointestinal, cardiovascular, respiratory, urogenital) with autonomic complaints, as well as exhaustion/fatigue (5).

The triple term “non-specific, functional and somatoform (NFS) bodily complaints” is primarily used to describe a group of disorders or diseases for which no clear organic cause can be identified and which are due to a dysfunction or interaction between different organs or systems of the body (5, 6). This term combines the terms “non-specific,” “functional” and “somatoform” and refers to disorders or diseases with physical symptoms or complaints for which no organic cause is found (7, 8). Accordingly, NFS can be considered when the following three criteria are met: (a) the complaints cannot be attributed to a specific disease; (b) the function of the affected organ or organ system is impaired; (c) the complaints significantly affect daily life (5). Patients with NFS are common in general practice (5, 6). By definition, NFS does not have a clear organic cause. However, it is not possible to say whether the cause of the complaints is psychological or biological (5). Irrespective of this, however, patients with NFS often report significant impairments in their psycho-behavioral functioning. The combination of somatic symptoms and dysfunctional psychobehavioral coping strategies, regardless of their etiology, can lead to significant social and occupational impairment and reduced quality of life (9, 10).

Against this background, the fifth edition of the Diagnostic and Statistical Manual of Mental Disorders (11) introduced the novel concept of “somatic symptom disorder (SSD).” This new disease conceptualization focuses on distressing somatic symptoms (so-called “A criterion”) and excessive and disproportionate thoughts, feelings, and behaviors associated with the somatic symptoms (so-called “B criteria”). Furthermore, the state of being symptomatic must have been persistent for at least 6 months (“C criterion”). Different from the NFS concept, the cause of symptoms is irrelevant in confirming the diagnosis of SSD—as long as thoughts, feelings and behaviors are incongruent with the cause.

Recent studies indicate that SSD is a common condition in general practice: the prevalence of SSD in the general adult population might be ~5–7% (11). According to Löwe et al. (12), SSD frequency rates, depending on the study population, sampling strategies, and diagnostic approaches applied in the single studies, varied from 3.5 to 77.7%. The mean frequency of SSD was 12.9% (95% CI, 12.5–3.3) in the general population and 35% (95% CI, 33.8–36.3) in general medicine settings. SSD is therefore a common problem, particularly in general medicine.

Despite SSD being one of the most frequent categories of patient complaints in primary care (13), the challenging diagnostic process is often underestimated (14). The complex clinical manifestation of SSD and the consistently delayed diagnoses result in frequent health care attendance (15). This can lead to unnecessary, invasive diagnostic tests or treatments (16). Dealing appropriately with high medical utilization patterns therefore poses a major challenge to primary care physicians (17) and financially strains the healthcare system through heightened expenditures (15, 16, 18).

Therefore, primary care physicians (PCPs) play an important role in identifying and managing SSD, and effective instruments for detecting SSD in primary care are needed. In recent years, several self-administered questionnaires have been developed as brief screening tools for a limited period to identify patients at risk for SSD (19–21). Two of the most established questionnaires are the 8-item Somatic Symptom Scale (SSS-8) and the 12-item Somatic Symptom Disorder—B Criteria Scale (SSD-12).

While the SSS-8 could be used to assess symptom burden (22, 23), the SSD-12 focuses directly on the psychobehavioral aspects of SSD (24). These instruments have been validated in different healthcare settings and among various patient populations (13, 21, 22, 24, 25). Conditions such as chronic somatic illness and mental health disorders (depression and anxiety) have also been included in validation studies (13, 21).

However, while the SSS-8 focuses on the burden of symptoms during the past 7 days, the duration of symptoms is not specified in the SSD-12. When a patient with a simple acute infection (e.g., common cold, gastroenteritis) presents to primary care, it is also unclear whether these two screening questionnaires are still effective in differentiating between simple acute infections and somatic symptom disorders.

The overall aim of our study was to investigate how the presence of a simple acute infection affects symptom burden in the two established questionnaire instruments SSS-8 and SSD-12. To this end, we explored whether there were differences between patients with a simple acute infection and patients with chronic somatic symptoms in terms of symptom burden assessed with the SSS-8 and psychobehavioral burden assessed with the SSD-12. We postulated that patients with a simple acute infection and patients with chronic somatic symptoms have a similar somatic symptom burden, but the patients with chronic somatic symptoms a higher psychobehavioral burden in comparison with the patients with a simple acute infection.

## 2. Methods

### 2.1. Study design

This study was part of a large cross-sectional, multicenter case–control study on functional gastrointestinal disorders funded by the Köhler Stiftung and registered in the German Clinical Trials Register (DRKS00011685). Ethics approval was obtained by the University of Heidelberg Ethics Committee prior to the commencement of subject recruitment. For this study, all eligible participants were asked to complete questionnaires about physical symptoms and mental health. In addition to self-report surveys, diagnostic data were collected from the treating physician. No patient-related data collected *via* the surveys were shared with the treating physician.

### 2.2. Sample

Study participants were recruited by a medical doctoral student in two primary care practices in Heidelberg, Germany between February and May 2017. Each patient entering the practice was asked if they would be willing to take part in the study. Informed consent and German language sufficiency were requirements for participation. Excluded from the study were patients (1) under 18 years of age, (2) suffering from acute psychosis or other severe psychiatric or somatic comorbidity that made participation in the assessment impossible, and (3) visiting the practice for reasons other than physical or mental health complaints.

### 2.3. Measures

The sociodemographic, somatic and psychological characteristics were assessed by a questionnaire set that all patients completed before they were seen by a physician.

#### 2.3.1. Questionnaire for sociodemographic data

Sociodemographic data were obtained *via* a modified version of the PsyBaDo questionnaire. The original PsyBaDo questionnaire, a well-established instrument in psychotherapy practice, facilitates the standardized collection of sociodemographic data, current symptoms, therapy goals (26), and assures comparability of data and quality in practice and research. The level of education was quantified using the International Standard Classification of Education (ISCED) scale (27), in which 10 education levels are classified from 0 to 9. A higher level on the scale correlates with a higher education level.

#### 2.3.2. Questionnaires for evaluation of somatic and psychological distress and medical conditions

##### 2.3.2.1. Somatic Symptom Scale-8

The eight-item was originally developed as an abbreviated version of the Patient Health Questionnaire-15 (PHQ-15) (28) and validated in the general population (22) as well as inpatient and outpatient settings (23) to detect somatic symptom burden. A 5-point Likert-scale (0 = not at all, 4 = very strongly) assesses the level of eight somatic symptoms experienced in the past seven days. According to the sum score, the burden can be categorized into minimal (0–3), low (4–7), medium (8–11), high (12–15), and very high (16 and above) (22). Therefore, a cutoff score of 8 was used in our study to indicate moderate somatic symptom burden.

##### 2.3.2.2. Somatic Symptom Disorder-12

This 12-item questionnaire quantifies the extent of psychological distress related to somatic symptoms. This instrument was developed by Toussaint et al. (24) considering the introduction of SSD and comprised three psychological B-criteria of SSD in three subscales: (1) cognition; (2) affect; and (3) behavior. Each subscale is measured by four items on a 5-point Likert-scale (0 = never to 4 = very often), with higher sum scores depicting greater distress. It has been validated in psychosomatic outpatient settings (24), the general population (21), and primary care (13). A cutoff score of 18 was chosen to screen for potential SSD cases at the time of our study after communication with the research team of SSD-12.

##### 2.3.2.3. Patient Health Questionnaire-9

This nine-item self-administered survey with a 4-point Likert-scale (0 = never to 3 = every day) measures the severity of depression. A higher sum score indicates more severe depressive symptoms (29). The Patient Health Questionnaire-9 (PHQ-9) has been validated in primary and secondary care settings and found to have a sensitivity and specificity of 88%, respectively, when using total scores ≥10 as the cutoff point for predicting major depression (29). In our study, the PHQ-9 was included in the screening module to gauge depression severity of participation.

##### 2.3.2.4. Generalized Anxiety Disorder-7

The Generalized Anxiety Disorder-7 (GAD-7) was included as a screening tool for assessing the level of generalized anxiety disorder (30). This seven-item questionnaire was developed specifically in the primary care setting and has been validated accordingly (31). The Likert-scale (0 = never to 3 = every day), assessing symptoms of the past 2 weeks, yields a sum score from 0 to 27 points, with higher scores representing greater anxiety levels. A sum score of ≥10 is the cutoff point for detecting a generalized anxiety disorder (sensitivity of 89% and specificity of 82%) (31).

##### 2.3.2.5. Chronic Condition Indicator

This study utilized a modified version of the Chronic Condition Indicator (CCI), which was initially developed as part of the Health care Cost and Utilization Project (HCUP) of the US Agency for Health care Research and Quality (32). The survey facilitates standardized documentation within medical practice and research by allowing subjects to self-report their chronic medical conditions. In addition to 19 somatic conditions, we added four psychosomatic syndromes and six mental illnesses to this survey for maximal informational gain. In each category, patients could also add any condition that was not listed in a field labeled “other.” The CCI total score directly reflects the number of chronic conditions listed.

##### 2.3.2.6. Simple acute infection

The presence of a simple acute infection was identified *via* participant self-reporting using the question “Have you suffered from a simple acute infection (e.g., common cold, severe cold or gastroenteritis) in the last seven days?” and the main symptoms that the patient reported.

#### 2.3.3. Physician's diagnostic assessment

After screening, all patients meeting at least one of the following inclusion cutoffs underwent a diagnostic assessment by their treating physician: (a) SSS-8 ≥ 8, (b) SSD-12 ≥ 18, (c) PHQ-9 ≥ 10, and (d) GAD-7 ≥ 10. Using cutoff points prevented the inclusion of symptom-free patients in the study. However, they were not used as a basis for subsequent group allocation. A careful selection process combining patient self-report and physician assessment was used as the basis for subsequent group allocation. Patient self-report was used for the assessment of acute infections and specific chronic diseases, while physician assessment was used for the assessment of non-specific functional symptoms. To ensure accuracy, participating physicians were given specific instructions to make a definitive nosological classification of symptoms, including the presence or absence of NFS. If present, they were instructed to make an accurate diagnostic classification of the specific NFS (e.g., irritable bowel syndrome or irritable stomach syndrome). This assessment was based on the physician's knowledge of the patient's medical history, symptoms, clinical examination and any relevant diagnostic tests. Physicians could note if a patient was new and thus difficult to evaluate or if the diagnosis was unclear for other reasons. Any other information from the patient's survey was not shared with the treating physician. The physician's diagnostic classification was used to verify the presence of an NFS diagnosis in the patient and for subsequent group allocation.

#### 2.3.4. Group definition

To examine how the presence of a simple acute infection affects complaint burden in the two established questionnaire instruments, SSS-8 and SSD-12, patients with a simple acute infection were compared with patients to somatic symptoms that were not due to infection. For this purpose, patients were assigned to one of the following two groups depending on the presence of a simple acute infection and the presence/absence of a chronic disease state. Acute Infection Group (AIG): all patients were included who (a) affirmed the presence of a simple acute infection and (b) had neither a chronic somatic nor functional disorder. Somatic Symptoms Group (SSG): Here, all subjects were included who (a) affirmed the presence of at least ≥ 1 chronic somatic disease or were identified with somatoform/functional disorder, and (b) in whom no acute infection was present.

The presence of chronic somatic diseases was assessed in a structured manner according to the CCI; the diagnosis of somatoform/functional disorder was verified by the diagnostic judgment of the responsible practitioner. In order to be consistent with the concept of SSD, patients with NFS and those with chronic somatic disease but without acute medical need for treatment were included in the Somatic Symptoms Group (SSG).

### 2.4. Statistical analyses

All statistical analyses were carried out by IBM SPSS Statistics 26.

A between-patient comparison was carried out to assess the effect of simple acute infections (independent variable) on SSS-8 and SSD-12 scores (dependent variables). Due to a violation of normality assumptions for both measures, confirmed *via* Shapiro–Wilks test, non-parametric Mann–Whitney *U* tests were carried out to compare the difference between the total score and the subscales of SSS-8 and SSD-12 between the two groups. Bonferroni corrections were carried out across the two main outcomes, SSS-8 and SSD-12, leading to a corrected alpha level of α = 0.025. To investigate which items were responsible for putative group differences, lower-order comparisons for individual items were carried out using the same analysis procedure. No correction for multiple testing was carried out on the item level. In this context, the *p*-value was used as a complement to the provided effect sizes. Effect sizes were calculated as Cohen's d. To illustrate the effect of simple acute infections on potential diagnostic validity, the chi-square test was used to compare the fraction of patients who met the cutoff points of the SSS-8 and SSD-12 between the two groups (α = 0.025).

## 3. Results

### 3.1. Participation

**Figure 1:**
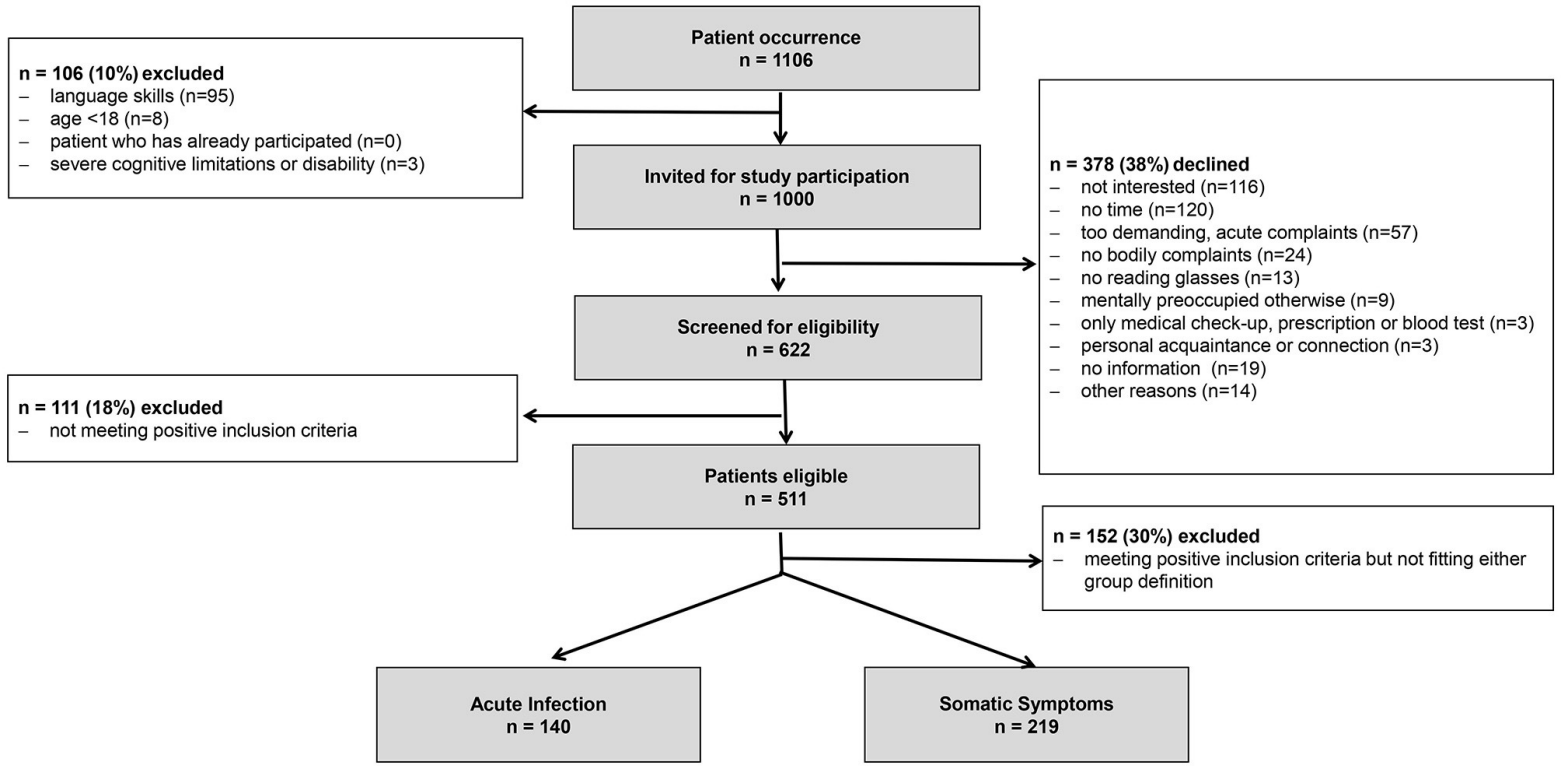
Flowchart of sample recruitment.

As shown in Figure 1, of 1,106 patients who visited two primary care practices, 1,000 were invited to participate in the study. A total of 378 patients declined, resulting in a participation rate of 62.2%. Of those the patients who accepted to participate, 82.2% met at least one of the prerequisite criteria (cutoff points) and were eligible to be included in the study. An overall recruitment rate of 46.2% (511/1,106) was achieved.

### 3.2. Sociodemographic characteristics and severity of depression and anxiety

Table 1 shows the sociodemographic characteristics of our patient sample. While the average age, rate of international patients, and living arrangements were similar between the two study groups, there were fewer female patients in the AIG, and the participants had a higher educational level than in the SSG.

**Table 1:** Sociodemographic characteristic and severity of depression and anxiety.

	**Acute infection (*n* = 140)**	**Somatic symptoms (*n* = 219)**	***P* (2-sided)**
**Age (mean** **±SD)**	33.61 (± 11.89)	37.30 (± 14.75)	0.054
**Sex female (** ***n*** **, %)**	68 (48.6%)	136 (62.1%)	0.009
**Nationality German (** ***n*** **, %)**	128 (91.4%)	195 (89.0%)	0.715
**Educational level ISCED** ** < 2 (*****n*****, %)**^**a**^	15 (10.7%)	45 (20.6%)	0.013
**Living situation**			
Alone (*n*, %)	36 (25.7%)	61 (27.9%)	
With partner (*n*, %)	41 (29.3%)	55 (25.1%)	
With children (*n*, %)	3 (2.1%)	12 (5.5%)	0.658
With partner and children (*n*, %)	26 (18.6%)	37 (16.9%)	
Other living arrangements (*n*, %)	34 (24.3%)	54 (24.7%)	
**PHQ-9 (mean** **±SD)**	4.80 (± 3.87)	7.58 (± 6.20)	<0.001
**GAD-7 (mean** **±SD)**	2.74 (± 3.10)	5.57 (± 4.97)	<0.001

a ISCED level 2: lower secondary education.

In addition, the severity of depression and anxiety is also demonstrated in Table 1. The group with simple acute infections showed a lower sum score than the group with somatic symptoms, with an overall medium effect size [PHQ-9 (d = 0.51, *p* < 0.001), GAD-7 (d = 0.65, *p* < 0.001)]. PHQ-9 (Cronbach's α = 0.869) and GAD-7 (Cronbach's α = 0.905) showed good internal reliability in our study.

### 3.3. Comparison of SSS-8

The group with simple acute infections (7.10 ± 5.04) showed a lower total score than the group with somatic symptoms (8.63 ± 5.90), with an overall small effect size (d = 0.27, *p* = 0.026). Cronbach's α for internal reliability of SSS-8 was 0.762 in this study.

**Figure 2:**
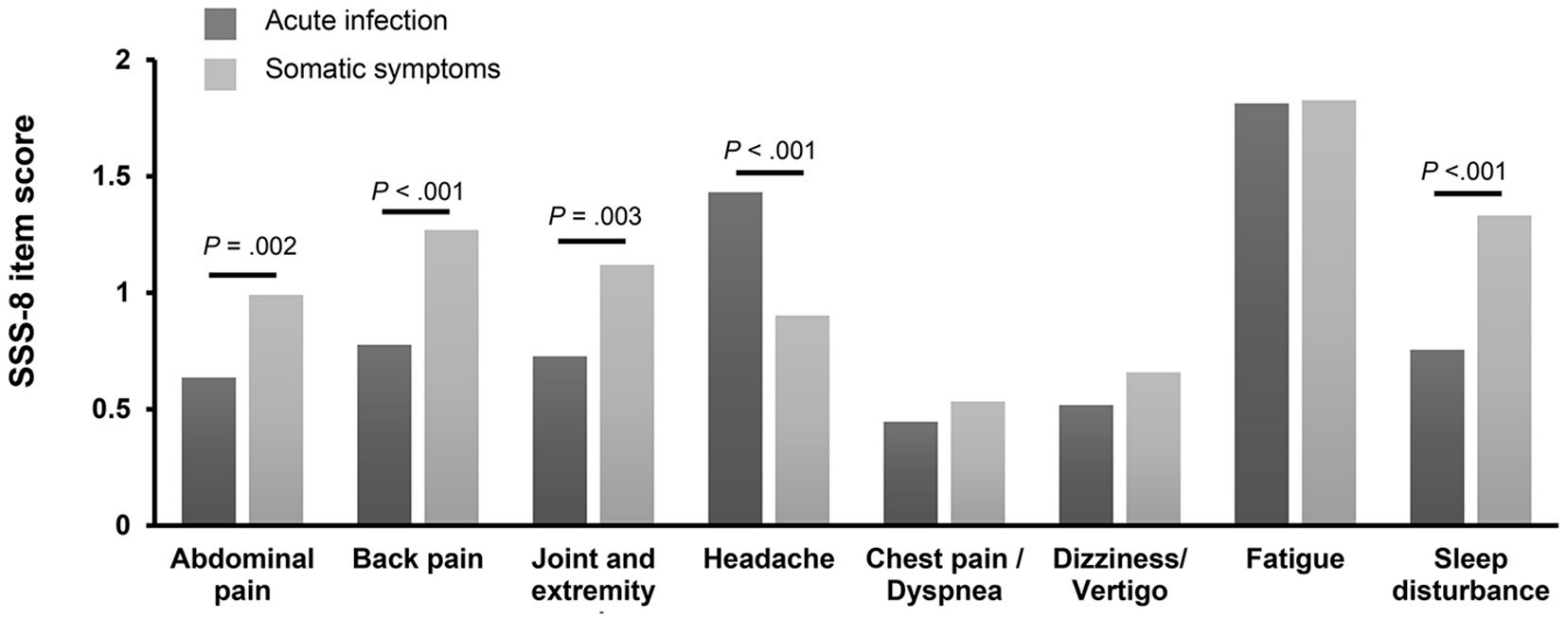
Comparison of each SSS-8 item score stratified according to symptoms. The figure shows the scores (0 = not at all, 4 = very strongly) for the different symptom dimensions of the Somatic Symptom Scale-8 (SSS-8) stratified for participants of the acute infection group (*n* = 139) and participants of the somatic symptom group (*n* = 214).

Figure 2 shows the comparison of the mean score of each symptom of SSS-8 between the two groups. While headache was experienced more intensely by those in the AIG, patients in the SSG experienced more severe abdominal pain, back pain, joint and extremity pain, and sleep disturbance. There was no difference in the severity of chest pain (d = 0.09, *p* = 0.566), dizziness (d = 0.15, *p* = 0.187), or fatigue (d = 0.01, *p* = 0.787) between the two groups. A comparison of the different symptom prevalence within the groups showed that fatigue was experienced most intensely among all symptoms in both groups.

### 3.4. Comparison of SSD-12

**Figure 3:**
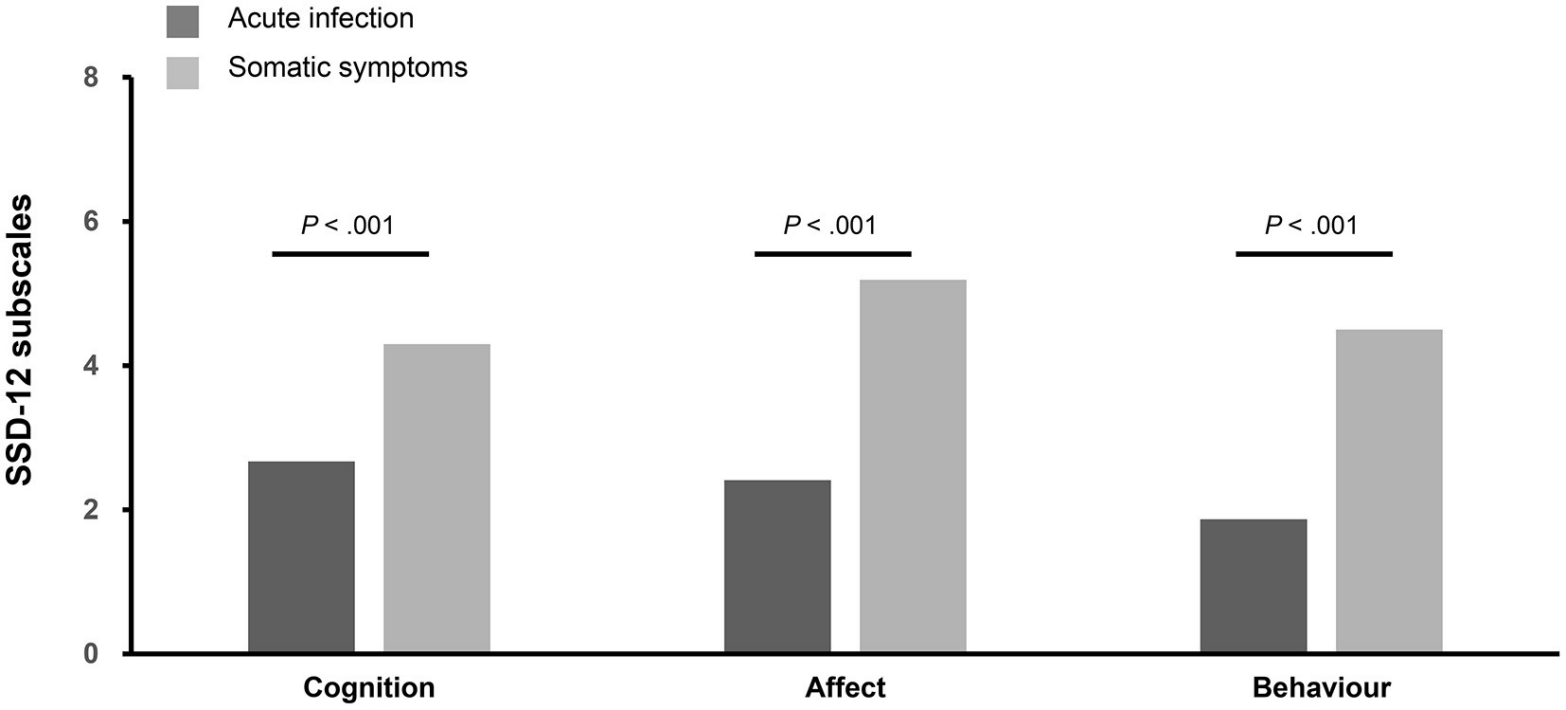
Comparison of the three SSD-12 subscales. The figure shows the different subdimensions (0 = not at all, 4 = very strongly) of the Somatic Symptom Disorder-12 (SSD-12) questionnaire stratified for participants of the acute infection group (*n* = 138) and participants of the somatic symptom group (*n* = 214).

The patients in the AIG showed a lower mean sum score (d = 0.76, *p* < 0.001) of SSD-12 (6.95 ± 6.72) than the patients in the SSG (13.99 ± 10.60). As shown in Figure 3, the mean scores of the cognition (d = 0.58), affect (d = 0.77), and behavior (d = 0.70) subscales in the AIG were also lower than the corresponding scores in the SSG. The SSD-12 showed excellent reliability (Cronbach's α = 0.931) in our study.

### 3.5. Comparison of the proportion of patients meeting the cutoff value of the SSS-8 and SSD-12

**Figure 4:**
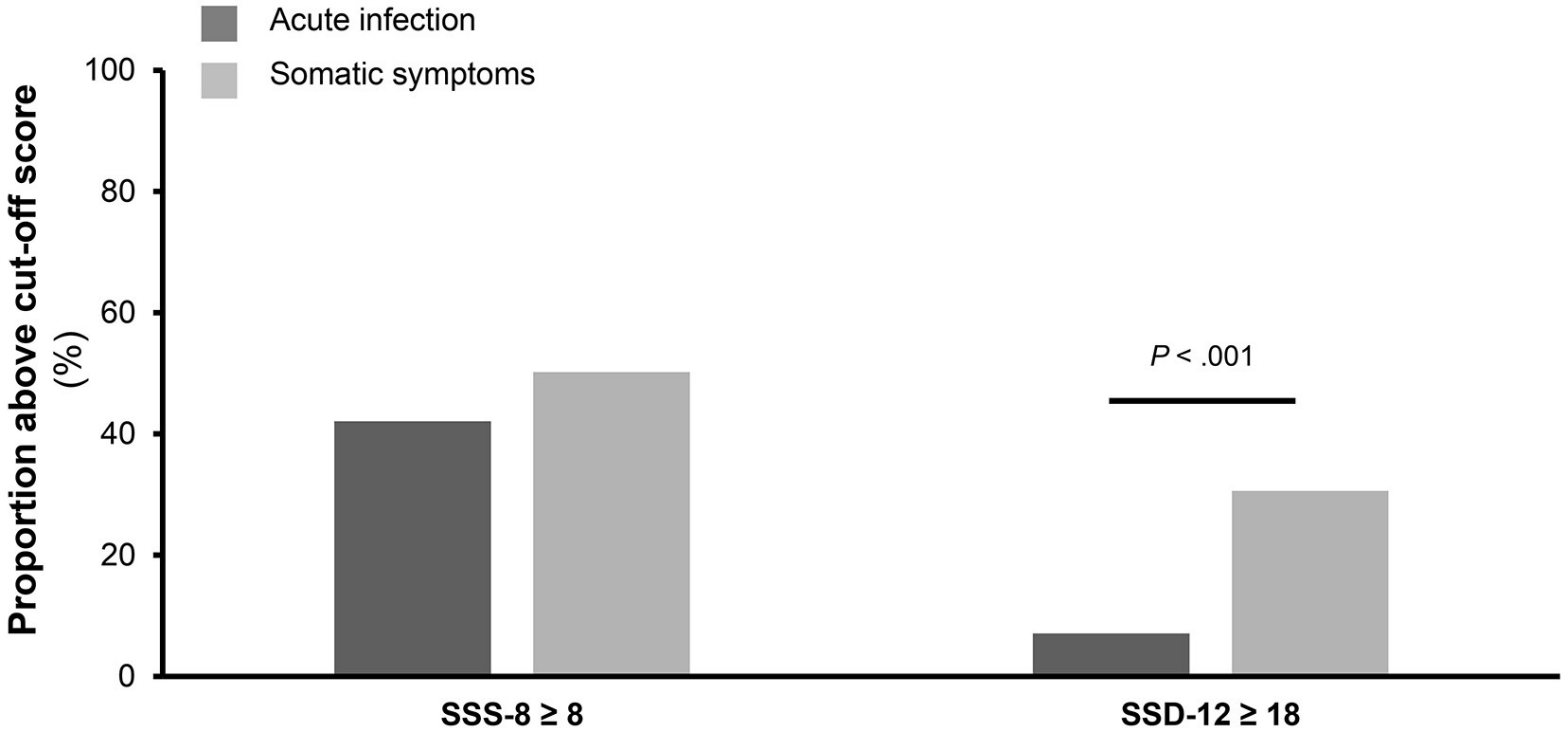
Comparison of percentage of participants meeting the cutoff values. The figure shows the of percentage of participants meeting the cutoff values of the Somatic Symptom Scale-8 (SSS-8) and Somatic Symptom Disorder-12 (SSD-12) questionnaire stratified for participants of the acute infection group (*n* = 140) and participants of the somatic symptom group (*n* = 214).

While the percentage of patients reaching the SSS-8 cutoff score of 8 out of 32 points did not differ between the two groups (42.1 vs. 50.2%), there were more patients in the SSG meeting the SSD-12 cutoff of 18 out of 48 points (*p* < 0.001). Overall, nearly half of the patients reported above-threshold severity of somatic symptoms in each group, whereas 7.1% of the patients in the AG and 30.6% of the patients in the SSG had above-threshold psychobehavioral distress (see Figure 4).

## 4. Discussion

This cross-sectional study aimed to investigate how the presence of a simple acute infection affects the complaint burden in the two established questionnaire-based screening instruments SSS-8 and SSD-12. For this purpose, we compared the somatic and psychobehavioural symptom burden in the context of a simple acute infection with individuals with chronic somatic complaints using these two established questionnaire instruments.

Although there were differences between the groups in both SSS-8 and SSD-12 total scores, the SSS-8 was more susceptible to changes triggered by symptoms of simple acute infections than the SSD-12. These results suggest that the SSD-12 is less susceptible to inflation by symptoms of simple acute infections and that its total score and corresponding cutoff value provide a more specific and thus practical tool for detecting SSD in primary care.

The significantly stronger dependence of the SSS-8 is not surprising since the SSS-8 primarily focuses on diffuse physical symptoms, which are a common accompaniment of simple acute infections. However, although this bias is obvious and hardly questioned, it has so far not been systematically explored nor its magnitude investigated. Our study demonstrates that the symptoms of headache and fatigue are difficult to screen for SSD in patients with simple acute infections. Even though this is well-known, it has rarely been considered in clinical practice. It seems simple and obvious to specify the instructions to exclude those patients who report symptoms of a simple acute infection.

In contrast to the SSS-8, the SSD-12 focuses on cognitive and behavioral factors, which are also influenced in the context of simple acute infections but are not directly associated with the acute infections. Our data show that this new approach focusing on symptom-related psychobehavioral distress seems promising. Compared to the AIG, patients in the SSG were characterized by both higher SSD-12 total scores and higher scores on the individual subscales, i.e., they suffered from more excessive thoughts, feelings, and behaviors related to disruptive somatic symptoms. Interestingly, the differences between the AIG and SSG groups showed equal magnitude on all three subscales, each with large effect sizes, which also suggests this screening tool is very robust.

Our data suggest that the sole assessment of somatic symptom burden with the SSS-8 in primary care is not specific enough for screening for SSD. Rather, it is important to take symptom-related psychobehavioral distress into consideration when facing patients with somatic symptoms in a setting with a high proportion of acute infections. The SSD-12 is thus a more effective instrument for detecting and assessing these conditions. Similar findings were obtained in the study of Toussaint et al. (13). They found that patients with self-reported chronic disease or/and at least one psychological disorder described a higher level of psychological burden experienced through their somatic symptoms than patients without self-reported chronic disease or/and psychological disorder. As a result, the SSD-12 was found to be appropriate to differentiate between the respective patient groups. The different proportions of patients who met or exceeded the cutoff points for SSS-8 and SSD-12 in the two groups also confirmed this point. While the percentage of patients with values equal to or greater than the SSS-8 cutoff values was almost equal between the two groups (42% in AIG vs. 50% in SSG), there was a significantly greater percentage of patients meeting or surpassing the diagnostic threshold (7% in AIG vs. 30% in SSG) of SSD-12 in the somatic symptom group. This indicated that several patients experienced severe somatic symptoms without distinct psychobehavioral distress. Screening solely for symptom severity without evaluation of psychological burden may lead to overdiagnosis of SSD in questionnaire-based studies.

Moreover, it should not be ignored that 7% of patients with simple acute infections reached or surpassed the cutoff points of the SSD-12. Even though this is a small proportion, the result suggests that a subgroup of these patients suffer from enormous psychological distress and might be at risk of developing SSD (33). However, this is speculative, and future studies are needed to answer this hypothesis.

## 5. Limitations

There were several limitations to this study. First, the identification of NFS bodily complaints in our study relied on the clinical evaluation of the treating physicians in primary care instead of standardized diagnostic interviews. Some PCPs had difficulties in clearly assessing the presence of a somatoform/functional disorder in individual patients because the symptomatology was very complex and their knowledge about some patients was too limited. However, we had explicitly selected GP practices that had many years of experience in this area as well as specific additional training in primary psychosomatic care in the past. The method being discussed here may not be as sensitive or specific as a structured clinical interview. This is because it can be affected by interrater errors. However, this method reflects how non-specific functional bodily complaints are detected in primary care. PCPs seldom use structured diagnostic tools, but instead rely on their intuition, long-term familiarity with a patient's medical history, and habitual decision-making. (34). For future studies, a preliminary training session regarding SSD and its diagnostic criteria may help standardize the diagnostic procedure. Second, it should be considered that for the diagnosis of SSD, the criterion that persistence of complaints should be longer than 6 months must be fulfilled. Therefore, a temporary simple acute infection does not usually meet SSD criteria. However, our study did not aim to investigate the diagnosis by questionnaire but the use of these instruments in primary care for screening purposes as it is applied in the daily clinical routine. The time criterion has so far not been sufficiently taken into account in these questionnaire-based approaches.

## 6. Conclusion

Our study shows that a simple acute infection significantly affects commonly used questionnaire-based screening instruments for the presence of SSD. The SSD-12 is less susceptible to symptoms of simple acute infections compared to the SSS-8 and thus provides a more specific and practical screening tool for identifying SSD in primary care. Even though this fact is obvious, it has not been systematically explored so far and has rarely been considered in clinical practice. With this in mind, our study helps to raise awareness in this area.

## Data availability statement

The original contributions presented in the study are included in the article/supplementary material, further inquiries can be directed to the corresponding author.

## Ethics statement

The studies involving human participants were reviewed and approved by Ethics Research Committee of the Faculty of Medicine, University of Heidelberg. The patients/participants provided their written informed consent to participate in this study.

## Author contributions

DB, MS, SB, AG, WE, and JT conceived and designed the study. SB obtained funding. MS, SB, AG, and JT collected the data. DB and MS statistically analyzed the data and all authors interpreted the data. YZ, MS, and JT drafted the manuscript. YZ is acting as the submission's guarantor. This manuscript and the data are based on the doctoral thesis of MS “Screeninginstrumente zur Diagnostik von funktionellen Störungen in derHausarztpraxis—Einfluss akuter Infekt” under the supervision of JT. All authors critically revised the manuscript and provided important intellectual content. All authors approved the final version of the article, including the authorship list.
